# Interdisciplinary Management of Cystic Neoplasms of the Pancreas

**DOI:** 10.1155/2012/513163

**Published:** 2012-10-21

**Authors:** Linda S. Lee, Thomas Clancy, Vivek Kadiyala, Shadeah Suleiman, Darwin L. Conwell

**Affiliations:** ^1^Interdisciplinary Management of Pancreatic Cystic Tumors (IMPACT) Clinic, Fish Center for Women's Health, Brigham and Women's Hospital, Harvard Medical School, Boston, MA 02115, USA; ^2^Center for Pancreatic Disease, Division of Gastroenterology, Hepatology, and Endoscopy, Brigham and Women's Hospital, Harvard Medical School, Boston, MA 02115, USA; ^3^Department of Surgery, Brigham and Women's Hospital, Harvard Medical School, Boston, MA 02115, USA

## Abstract

Cystic neoplasms of the pancreas are increasingly recognized due to the frequent use of abdominal imaging. It is reported that up to 20% of abdominal cross-sectional scans identify incidental asymptomatic pancreatic cysts. Proper characterization of pancreatic cystic neoplasms is important not only to recognize premalignant lesions that will require surgical resection, but also to allow nonoperative management of many cystic lesions that will not require resection with its inherent morbidity. Though reliable biomarkers are lacking, a wide spectrum of diagnostic modalities are available to evaluate pancreatic cystic neoplasms, including radiologic, endoscopic, laboratory, and pathologic analysis. An interdisciplinary approach to management of these lesions which incorporates recent, specialty-specific advances in the medical literature is herein suggested.

## 1. Introduction

With improvements in abdominal radiologic imaging, incidental pancreatic cystic neoplasms are increasingly discovered in as many as 20% of patients undergoing computed tomography (CT) or magnetic resonance imaging (MRI) for nonpancreatic indications. The proper management of these lesions is critical [1–5]. 

The differential diagnoses for incidental pancreatic cystic lesions include the following: (1) benign serous cystadenoma or (2) premalignant mucinous cystic lesions, which are categorized into mucinous cystic neoplasm (MCN), branch duct intraductal papillary mucinous neoplasm (BD-IPMN), main duct IPMN (MD-IPMN), or mixed IPMN. Based on the histologic type, pancreatic cystic neoplasms may have low or high risk for malignant transformation. 

Unfortunately, the imaging characteristics of pancreatic cysts can be similar, making differentiation between benign and premalignant conditions difficult. In addition, current cyst fluid analysis techniques fail to clearly distinguish among the cysts. The definitive classification of pancreatic cysts is crucially important since precancerous lesions may require surgical resection, while others that are benign or indolent can be observed. 

An interdisciplinary approach incorporating medical pancreatology, therapeutic endoscopy, and pancreatic surgery is critical to the evaluation of patients with cystic neoplasms of the pancreas. We provide a brief overview of the clinical problem followed by interdisciplinary management algorithms based on the current literature, including recent guidelines from the major gastrointestinal and surgical societies.

## 2. Clinical and Pathologic Features 

Unlike most hepatic and renal cysts, pancreatic cystic lesions raise clinical concern because of their potential for malignancy and malignant transformation. About 50–60% of pancreatic cystic lesions are cystic neoplasms, while cystic degeneration of solid neoplasms represents 10%, and pseudocysts account for almost one-third of pancreatic cystic lesions. Nearly 90% of all pancreatic cystic neoplasms are benign serous cystadenomas and premalignant or malignant mucinous lesions, which include the parenchymal MCN or intraductal papillary mucinous neoplasm (IPMN). Appropriate management of these cystic neoplasms varies considerably among the various types; a review of the salient features of the most common lesions is outlined below (Table 1) [6–16]. 

*Serous cystadenomas (SCAs) *are benign pancreatic cystic neoplasms, which very rarely become malignant. They account for over 30% of pancreatic cystic neoplasms and arise anywhere in the pancreas. Some studies suggest higher incidence of SCA in the body and tail of the pancreas, while others favor the head and neck. SCA typically occurs in women over the age of 60. The natural history of SCAs is not well described; however, they appear to grow over time. One study suggested that cysts smaller than 4 cm expand at a slower rate (0.12 cm/year) than cysts larger than 4 cm (1.9 cm/year). Malignant transformation is extremely rare with only a few case reports of serous cystadenocarcinoma. On pathologic examination, SCAs are lined characteristically by glycogen containing cuboidal epithelial cells.

*Mucinous cystic neoplasms (MCNs)* are premalignant parenchymal lesions that occur almost exclusively in women between 40 and 50 years of age. They arise in the body and tail of the pancreas in approximately 95% of patients and are defined by the presence of ovarian-like stroma on pathology. Features concerning malignancy include older age, large size (especially >6 cm), and presence of thick cyst wall, mural nodules, or peripheral eggshell calcification. The true incidence of malignancy in MCNs is unknown although recent studies suggest lower rates of invasive cancer (12–29%) and carcinoma in situ (5.5%). 

*Intraductal papillary mucinous neoplasm (IPMN)* is also a mucinous cyst that arises from the pancreatic ductal epithelium of the main duct, side branches, or both. It occurs slightly more commonly in men between the ages of 50 and 60. While IPMNs usually arise in the head of the pancreas, they can occur anywhere in the pancreas as well as in multiple locations. There are *3 subtypes of IPMN:* main duct (diffuse or segmental involvement of the main duct, MD-IPMN), branch duct (dilation of one or more side branches, BD-IPMN), and mixed type (both main duct and side branch involvement, mixed IPMN). 

By pathology, IPMN may be classified as of gastric, intestinal, or pancreaticobiliary type. There are variable reports in the medical literature on the clinical significance of histological grading, whether some types may be more indicative of malignant potential. While histological grading may hold some predictive accuracy, this is currently only achievable postoperatively. 

Accurate differentiation among the clinical subtypes is important due to differences in malignant potential and management. MD-IPMN is characterized by dilation of the main pancreatic duct, usually due to a neoplasm in the proximal duct producing mucus that fills and dilates the entire duct, although it may also rarely result from tumor involving the entire duct. About 40% of MD-IPMNs contain malignancy at the time of diagnosis. Although main pancreatic duct dilation greater than 15 mm and presence of mural nodules have been associated with malignancy, malignancy is also present in up to 30% of patients with MD-IPMN without symptoms, mural nodules, or massive duct dilation. Alternatively in another study, 35% of MD-IPMN followed conservatively for a median of 48 months did not develop malignancy.

Approximately 15% of BD-IPMN can undergo malignant transformation. Ominous features suggesting progression in BD-IPMN include at least a 1 cm increase in cyst size, at least a 2 mm increase in main pancreatic duct size, or development of mural nodules over a median followup of 3.7 years, by one report. In this study, 32% of the patients demonstrating progression of BD-IPMN underwent surgical resection, and of these 31% were malignant.

Other less common pancreatic cystic neoplasms include *solid pseudopapillary neoplasm (SPEN)*, which occur almost exclusively in young women. SPENs account for 1-2% of pancreatic cystic neoplasms. They were first described in 1959 as Frantz or Hamoudi tumors and were renamed SPEN by the World Health Organization in 1996. Pathologic examination reveals characteristic pseudopapillae with cystic spaces containing hemorrhage and cholesterol clefts in myxoid stroma, alternating with solid tissue. SPENs may occur anywhere throughout the pancreas. About 10–15% of SPENs are malignant, and to date no predictors of aggressive behavior have been identified. In addition, less commonly observed lesions such as neuroendocrine or acinar cell tumors can sometimes undergo cystic degeneration.

## 3. Imaging Features and Cyst Fluid Analysis 

Differentiating among pancreatic cystic lesions and predicting malignant transformation can prove challenging. Current evaluation of suspicious pancreatic cystic neoplasms includes a combination of radiologic imaging, endoscopic ultrasound (EUS), and cyst fluid analyses. Overall, accurate diagnosis of pancreatic cysts by radiologic imaging occurs in about 40% to 60% of cystic lesions. CT and MRI both more accurately predict the presence of malignancy in pancreatic cysts (73% to 79%). These diagnostic rates are comparable to EUS imaging. Commonly observed imaging features are described below for the various cystic neoplasms [17–27]. 

**Figure 1:**
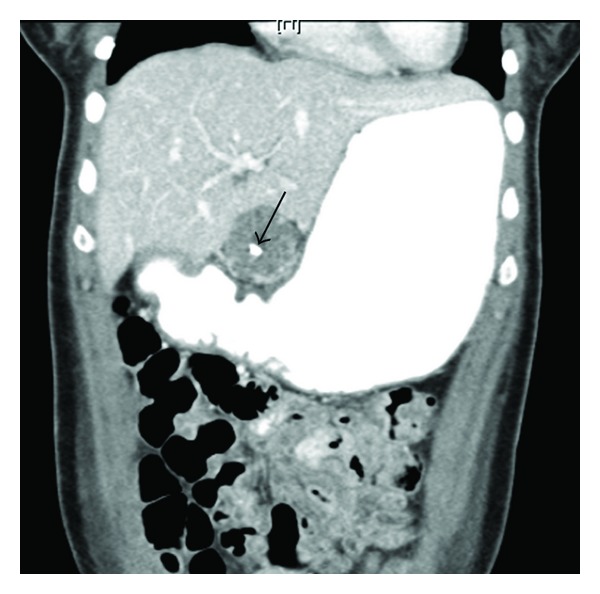
Serous cystadenoma with central scar (arrow) on abdominal CT.

Serous cystadenomas are typically multicystic with each cyst <2 cm and 30% have a lobular “honeycomb” appearance due to dense septations producing multiple small cysts. Up to 10% of SCAs may be unilocular or contain few septa making differentiation from MCN difficult. Occasionally these lesions may appear solid due to the presence of numerous microcysts that give the appearance of a homogeneous hypoechoic mass. The pathognomic central scar or “sunburst calcification” is present in only about 30% of SCA (Figure 1). 

**Figure 2:**
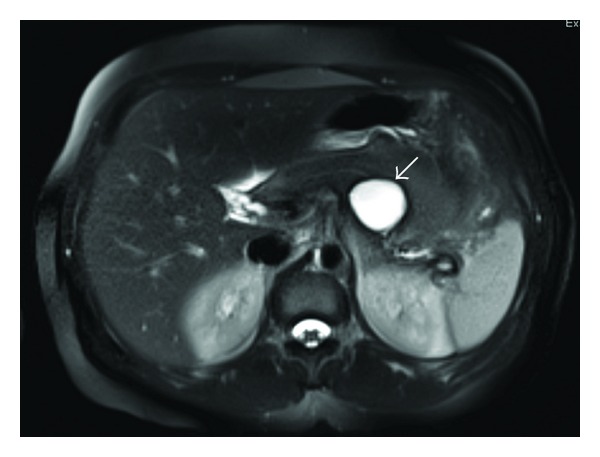
Mucinous cystic neoplasm (arrow) on MRI abdomen.

Unlike SCA, MCNs usually appear as smooth, well-defined, and unilocular or with only a few septations (Figure 2). Thick septae, mural nodules, and calcifications are features associated with malignancy. Calcifications within the peripheral wall of the cyst and occasionally within the cyst occur in less than 20% of MCNs. Without a clear history of acute or chronic pancreatitis, differentiation of MCN, even SCA and BD-IPMN, from pseudocysts may be difficult by imaging alone. Pseudocysts typically appear round or oval with a thin, enhancing, possibly calcified capsule on CT. Communication with the pancreatic duct may or may not be present.

**Figure 3:**
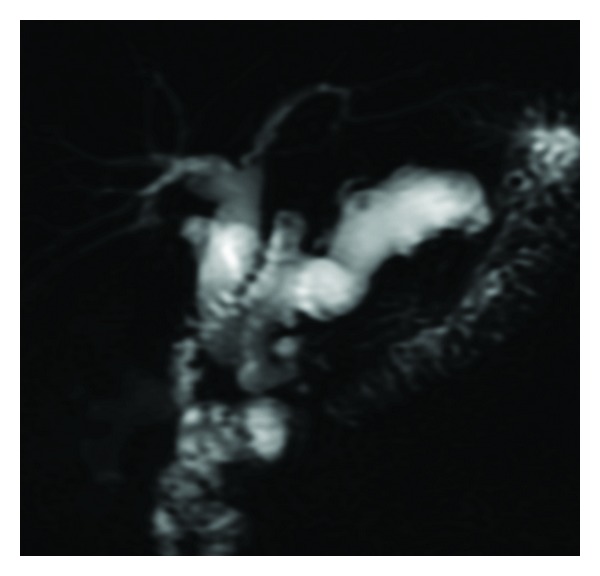
MD-IPMN with diffusely dilated main pancreatic duct on MRCP (magnetic resonance cholangiopancreatography).

**Figure 4:**
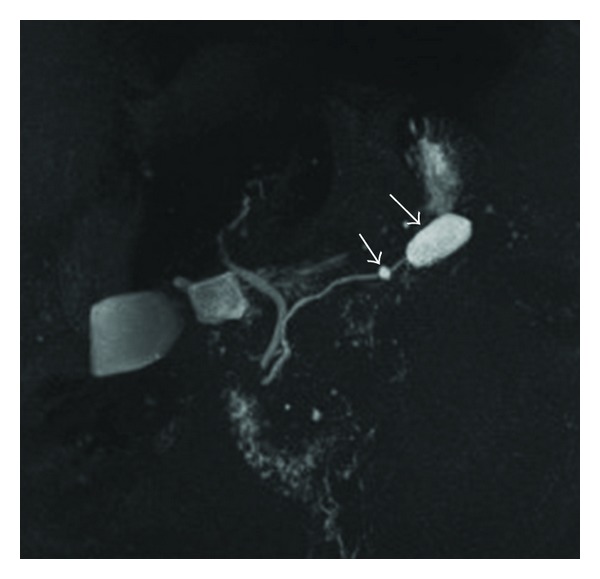
BD-IPMN with 2 cysts (arrows) communicating with nondilated main pancreatic duct on MRCP.

IPMNs are ductal lesions involving the main pancreatic duct, side branches, or a combination of both. MD-IPMNs lead to diffuse dilation of the main pancreatic duct (Figure 3). Diagnosis of BD-IPMN is predicated on demonstrating communication of the affected side branch with the main pancreatic duct (Figure 4). Approximately 20% of BD-IPMNs diagnosed by radiology are actually mixed IPMNs by pathology. This is clinically important because mixed IPMNs have a malignant potential similar to MD-IPMN, and, thus, surgical resection is recommended for these lesions.

Imaging findings concerning malignancy in BD-IPMN include main duct dilation greater than 10 mm and presence of a solid component or mural nodule. MRI identifies solid components, septa, main pancreatic duct dilation, communication with main pancreatic duct, and mural nodules with similar sensitivity to EUS. Both have modest sensitivity for detecting mural nodules (58% to 67%). Training in the detection of 3 EUS criteria (hypoechoic lesion, smooth edge, and hyperechoic rim) for mucus versus nodule improved diagnostic accuracy from 57% to 79%. 

**Figure 5:**
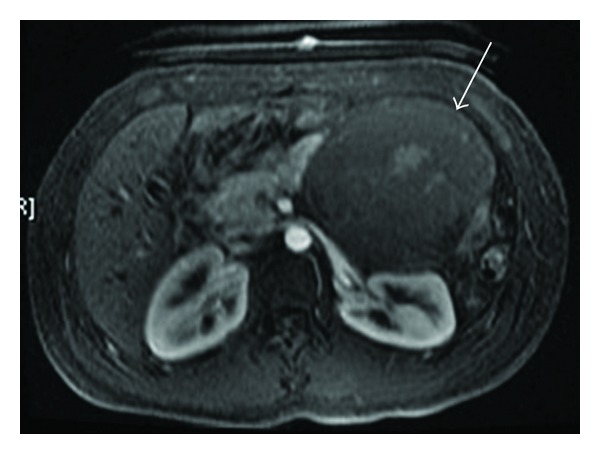
SPEN (arrow) with wall, internal septations and hemorrhage on MRI.

Unlike most other pancreatic cystic lesions, SPENs and cystic neuroendocrine tumors typically have characteristic findings on imaging. The rare SPEN usually presents as a large, well-defined, encapsulated mass with peripheral solid component and cystic degeneration in the center with areas of hemorrhage (Figure 5). Peripheral calcification is present rarely. Cystic neuroendocrine tumors account for about 10% of all pancreatic neuroendocrine tumors. They are highly vascularized with early enhancement of the rim during arterial imaging with MRI.

Cytologic evaluation of aspirated cyst fluid from fine needle aspiration (FNA) is often performed during EUS but at best has a sensitivity of 34% for mucinous lesions. Glycoprotein tumor antigens, such as carcinoembryonic antigen (CEA), are secreted by epithelium lining mucinous lesions and have been the focus of cyst fluid analysis. A CEA level >192 ng/mL has modest sensitivity (75%) and specificity (84%) for mucinous cystic. However, many mucinous lesions with CEA <192 ng/mL are missed using this cutoff. In addition, cyst fluid CEA level is not predictive of malignancy. Recent interest in DNA mutation analysis from cyst fluid has similarly proven disappointing. Measurement of allelic loss amplitude has a sensitivity of 67% and specificity of 66% for mucinous cystic lesions. The presence of a k-*ras* mutation is highly specific (96%) for mucinous lesions but has a low sensitivity of 45%.

## 4. Diagnostic Evaluation and Management

A diagnostic algorithm is proposed based on current data and review of medical literature. Current treatment guidelines from several major gastrointestinal societies recommend surgical resection for all definite MCNs and MD-IPMN. Since premalignant MCNs typically occur in younger women with a long life expectancy in the more easily accessible pancreatic tail, and malignancy is detected in about 70% of resected MD-IPMN specimens. Because the occurrence of malignancy is much lower in BD-IPMN (15–25% of patients), resection is reserved for those patients with pancreatitis symptoms, main pancreatic duct dilation >10 mm, presence of mural nodules, cytology suspicious or positive for malignancy, and/or possibly cysts >3 cm particularly in younger patients. Nonmucinous pancreatic lesions (serous cystadenomas) do not require further evaluation. The decision for surgical resection needs to be weighed based on age, comorbidities, and resectability. A recent Markov model incorporates these features to guide therapy. Furthermore, a white paper from the radiology community outlines an approach to asymptomatic cysts found on abdominal imaging [28–35]. 

Accurate diagnosis of “indeterminate” pancreatic cystic neoplasms is difficult and often not possible until surgical resection. For this reason we have developed an *Interdisciplinary Management of Pancreas Cystic Tumor (IMPACT) Clinic* that uses an initial triage algorithm that incorporates the current literature. As can be seen from Table 2, in *Step *1, clinical symptoms, laboratory data, and prior imaging findings are collected and reviewed by an IMPACT physician before the office visit. All patients must have a pancreas protocol CT scan and/or MRI/MRCP to evaluate the pancreas parenchyma and ductal anatomy. In *Step *2, office appointment triage is based on symptoms and definite diagnosis of high risk lesions, as outlined. In essence, all known MD-IPMN, MCN, and *symptomatic lesions* regardless of size are triaged initially for a surgical consultation to determine operability and resectability.

In *Step *3*, asymptomatic lesions* are triaged based on size. *Small, asymptomatic lesions* <1 cm in size, are initially seen by a medical pancreatologist and followed based on current IAP (International Association of Pancreatology) and radiology society guidelines. *Intermediate size lesions* (1–3 cm in size) are initially seen by a therapeutic endoscopist since EUS with cyst aspiration and analysis may be necessary for further characterization. *Large cystic lesions* >3 cm are initially seen by a pancreatic surgeon for consideration of surgical resection based on resectability and operability.

An intermediate *Step *4 is suggested if initial evaluation reveals *indeterminate findings *such as equivocal cyst fluid analysis, DNA markers, or borderline imaging features. This step involves a formal case presentation to the weekly *Multidisciplinary Pancreas Study Group* comprised of radiologists, pancreaticobiliary surgeons, therapeutic endoscopists, medical pancreatologists, and gastroenterologists. The case is presented by the IMPACT Clinic staff at which time a consensus opinion is given and later discussed with the patient and referring physician.

*Step *5 is a letter of communication from the IMPACT Clinic outlining the treatment plan. *Step *6 involves scheduling surveillance, surgical resection, or further imaging. Finally, patients are entered into a clinical database.

## 5. Conclusions

Pancreatic cystic neoplasms are increasingly recognized primarily due to the increased use and advancements of abdominal imaging. Current methodologies including imaging, endoscopy, and cyst fluid analysis are imperfect in reliably differentiating mucinous from nonmucinous pancreatic cystic lesions and also cannot predict malignant transformation with a high degree of accuracy. Strategies to manage this growing population of patients with potentially premalignant pancreatic cystic lesions are greatly needed, both to recognize lesions requiring aggressive surgical management, to avoid unnecessary surgeries, and to appropriately utilize diagnostic resources. Input from a variety of clinicians, including gastroenterologists, therapeutic endoscopists, radiologists, pancreatic surgeons, and pathologists, is vital. An interdisciplinary management algorithm is proposed that stratifies and triages patients based on symptoms, cyst size, and definitive diagnosis. 

## 6. Future Directions 

Better predictive biomarkers in cyst fluid are greatly needed and are under investigation. Recent data suggest that the presence or absence of GNAS mutations may help diagnose IPMN lesions. Gene expression profiling of pancreatic cyst fluid and confocal laser endomicroscopic examination of pancreatic cysts are novel techniques also currently being studied to better characterize lesions. In a recent meta-analysis, expression of hTERT is strongly associated with malignant transformation in IPMN, implicating upregulation of hTERT as a key step in progression of IPMN to cancer. Researchers are also using miRNA, proteomic and cytokine profiling of cyst fluid to discriminate among the premalignant and benign neoplasms. EUS with fine needle injection of alcohol into the cyst (alcohol ablation) with or without paclitaxel may be a viable option for cyst resolution in nonoperative candidates [17, 36–43]. 

## Figures and Tables

**Table 1:** Characteristics of common pancreatic cysts.

	Pseudocyst	IPMN	MCN	SCA
Gender (male : female)	1 : 1	2 : 1	0.5 : 9.5	1 : 4
Age range (yr)	40–70	60–80	30–50	60–80
Imaging features				
(i) Communication with main duct	Variable	Yes	No	No
(ii) Location	Any	Head/uncinate—50%	Body/tail—90%	Variable
Cyst fluid analysis				
(i) Amylase	High (>250 U/L)	High (>250 U/L)	Low (<250 U/L)	Low (<250 U/L)
(ii) Mucin	Low	High	High	Low
(iii) CEA (elevated: >192 ng/mL)	Low	Elevated	Elevated	Low
Malignant potential	No	Yes	Yes	No
Features suggestive of malignancy	None	Main duct > 10 mm, Branch duct: solid component, mural nodule, cytology suspicious or positive for malignancy	Larger than 6 cm, solid component, mural nodule	None
Incidence of invasive cancer (%)	0	MD-IPMN: 40–50 BD-IPMN: 15	12	Very rare
Treatment	Observation Resection if symptomatic	Resection: MD-IPMN Resection or surveillance: BD-IPMN based on presence of features suggestive of malignancy and comorbid disease	Resection	Resection if symptomatic

**Table 2:** Interdisciplinary management of pancreatic cystic tumors.

	IMPACT Clinic	Action points
*Step *1 Collect outside data for IMPACT MD to review before office visit	Initial review of outside medical records: symptoms, laboratory, imaging review with staff radiologist	Consider repeat pancreas protocol CT and MRI/MRCP to better visualize pancreatic parenchyma and ductal anatomy
		*Proceed to Step *2: arrange office visit and imaging based on symptoms and imaging

*Step *2 Initial triage based on symptoms or ominous features on imaging:	Symptomatic or high risk lesion	Surgical referral (age, ASA grade, resectability) may request EUS based on findings
recurrent pancreatitis, dilated main duct, mural nodule, solid component, obstructive jaundice, abrupt caliber change of duct	Asymptomatic	Triage based on size
		*Proceed to Step * 3

*Step *3 Secondary triage based on cyst size (i) IAP Guidelines, 2012 (ii) Am College of Radiology Guidelines, 2010 (iii) Markov model, 2010 (iv) Cost-effective analysis, 2009	Cyst <1 cm Cyst 1–3 cm Cyst > 3 cm	Medical pancreatology Therapeutic endoscopy for EUS Surgical referral (age, ASA grade, resectability) may request EUS based on findings
		*Proceed to Step *4* or *5 based on results of evaluation

*Step *4 Clinical challenges	Indeterminate results: equivocal imaging and/or cyst fluid analysis	Present case in weekly *Multidisciplinary Pancreas Study Group* for consensus recommendations
		*Proceed to Step * 5

*Step *5 IMPACT recommendations	Schedule follow-up appointment: surgical resection, EUS –FNA, or observation	Letter to referring MD and patient
		*Proceed to Step * 6

*Step *6 Followup	Surveillance recommendations based on imaging, fluid analysis, and/or surgical pathology findings	IMPACT Clinical Database entry Letter to referring MD and patient Automated follow-up letter: 3 mo, 6 mo etc…

Modified from Tanaka et al. [30], Berland [33], Khalid and Brugge [32], Das et al. [28], and Weinberg et al. [31].
